# An On-Treatment Decreased Trend of Serum IL-6 and IL-8 as Predictive Markers Quickly Reflects Short-Term Efficacy of PD-1 Blockade Immunochemotherapy in Patients with Advanced Gastric Cancer

**DOI:** 10.1155/2024/3604935

**Published:** 2024-05-14

**Authors:** Jiameng Liu, Yufei Mao, Chaoming Mao, Deqiang Wang, Liyang Dong, Wei Zhu

**Affiliations:** ^1^Department of Nuclear Medicine, The Affiliated Hospital of Jiangsu University, Zhenjiang, Jiangsu 212001, China; ^2^Department of Ultrasound Medicine, The Affiliated Hospital of Jiangsu University, Zhenjiang, Jiangsu 212001, China; ^3^Department of Oncology, Institute of Digestive Diseases, The Affiliated Hospital of Jiangsu University, Zhenjiang, Jiangsu 212001, China; ^4^School of Medicine, Jiangsu University, Zhenjiang, Jiangsu 212013, China

## Abstract

**Objective:**

Immunotherapy has proven effective in treating advanced gastric cancer (AGC), yet its benefits are limited to a subset of patients. Our aim is to swiftly identify prognostic biomarkers using cytokines to improve the precision of clinical guidance and decision-making for PD-1 inhibitor-based cancer immunotherapy in AGC.

**Materials and Methods:**

The retrospective study compared 36 patients with AGC who received combined anti-PD-1 immunotherapy and chemotherapy (immunochemotherapy) with a control group of 20 patients who received chemotherapy alone. The concentrations of TNF-*α*, IL-1*β*, IL-2R, IL-6, IL-8, IL-10, and IL-17 in the serum were assessed using chemiluminescence immunoassay at three distinct time intervals following the commencement of immunochemotherapy.

**Results:**

When compared to controls, patients undergoing immunochemotherapy demonstrated a generalized rise in cytokine levels after the start of treatment. However, patients who benefited from immunochemotherapy showed a decrease in IL-6 or IL-8 concentrations throughout treatment (with varied trends observed for IL-1*β*, IL-2R, IL-10, IL-17, and TNF-*α*) was evident in patients benefiting from immunochemotherapy but not in those who did not benefit. Among these markers, the combination of IL-6, IL-8, and CEA showed optimal predictive performance for short-term efficacy of immunochemotherapy in AGC patients.

**Conclusion:**

Reductions in IL-6/IL-8 levels observed during immunochemotherapy correlated with increased responsiveness to treatment effectiveness. These easily accessible blood-based biomarkers are predictive and rapid and may play a crucial role in identifying individuals likely to derive benefits from PD-1 blockade immunotherapy.

## 1. Introduction

Tumor immunotherapy represents a cutting-edge therapeutic avenue following conventional treatments like surgery, radiotherapy, and chemotherapy, showing promise in clinical applications [1]. However, its effectiveness varies widely among patients, posing a challenge in predicting responses. Through its interaction with programmed cell death ligand 1 (PD-L1), programmed cell death protein-1 (PD-1) functions as an immune system suppressor, preventing effector T cells from targeting cancer cells and promoting the growth of tumors [2]. PD-L1 expression in tumor tissue stands as the sole established biomarker for predicting immunotherapy response [3]. PD-1 blockade, a novel immunotherapy strategy, has gained widespread usage in treating diverse cancers, with several PD-1 inhibitor drugs approved by the Food and Drug Administration (FDA) for advanced gastric cancer (AGC) and other malignancies [4–6]. While the FDA approval disregards correct positive rate (CPS)/true positive rate (TPS) scoring, the European Medicines Agency's (EMA's) approval is contingent upon a specific threshold of PD-L1 expression in patients. Nonetheless, merely about 60% of patients achieve objective responses, underscoring the inadequacy of PD-L1 alone in forecasting response. Furthermore, heightened global scrutiny on healthcare costs and potential immunotherapy-related toxicities underscores the importance of precise patient selection. Despite the transformative impact of novel PD-1 inhibitors on therapeutic landscapes, an urgent need exists to ascertain the true efficacy of PD-1 blockade, given its limited benefit to a subset of patients due to disease and treatment response variations. Hence, prospective identification of patients likely to benefit from specific treatment regimens is imperative to mitigate risks of adverse clinical outcomes and soaring treatment expenses.

Presently, the evaluation of immunotherapy efficacy in clinical settings predominantly relies on monitoring tumor markers, imaging examinations, and the expression of immune checkpoint markers in tumor tissue [7–9]. Tumor markers offer insights into the activity status, quantity, and differentiation degree of tumor cells, yet their specificity and sensitivity are constrained, rendering them insufficient as standalone indicators of immunotherapy efficacy [10]. Imaging examinations enable direct observation of changes in tumor volume and morphology, providing valuable references for assessing treatment effectiveness. However, they entail limitations such as time delays, high error rates, and technical dependencies when evaluating immunotherapy efficacy [11, 12]. While immunohistochemical analysis of immune checkpoint markers in tumor tissue may help identify additional patients who could benefit from cancer immunotherapy, it presents numerous challenges in clinical application [13, 14]. Leveraging blood-based immune biomarkers can overcome the limitations associated with tissue-based biomarkers, as peripheral blood sampling is minimally invasive and blood-based tests can be repeated with ease [15].

From tumor onset and progression to therapeutic interventions, the immune response emerges as the earliest and most rapid factor. Cytokines, pivotal in regulating interactions between immune cells and other cellular components, serve as primary mediators in immune responses [16]. Within the cycle of “tumor initiation–immune recognition–tumor progression–immune alterations,” fluctuations in cytokine levels can either contribute to tumor initiation or arise as consequences of tumor-related changes [17]. Following this premise, dynamically monitoring changes in cytokine levels in peripheral blood emerges as a direct and efficient approach to assessing the short-term efficacy of immunotherapy. This study aims to pinpoint prognostic biomarkers derived from cytokine analysis, facilitating more precise clinical guidance and decision-making regarding PD-1 inhibitor-based neoadjuvant chemotherapy in AGC patients at the earliest possible juncture.

## 2. Materials and Methods

### 2.1. Patients and Study Design

We examined data from a cohort of 56 patients at Jiangsu University's Affiliated Hospital who were diagnosed with AGC between March 2022 and March 2023 in this retrospective analysis. The patients were divided into two groups: the experimental group, which included 36 AGC patients receiving immunochemotherapy, a combination therapeutic approach that included chemotherapy and anti-PD-1 monoclonal antibody (mAb) treatment, and the control group, which included 20 AGC patients receiving chemotherapy exclusively. Strict inclusion standards were implemented, necessitating the following: (1) According to the American Joint Committee on Cancer's gastric cancer (GC) staging guidelines, AGC is classified as stage III/IV clinically; (2) eligibility required patients to have undergone immunochemotherapy for a minimum of three treatment cycles, with an expected survival prognosis of over 3 months; (3) patients' Eastern Cooperative Oncology Group (ECOG) performance status scores were considered, with inclusion criteria limited to scores between 0 and 2 points, as specified in guideline reference [18]; and (4) thorough evaluations, encompassing routine blood analyses, assessments of liver and kidney functions, and electrocardiograms, were performed. It was imperative for the results of these evaluations to either remain within normal parameters or exhibit minimal deviations, devoid of any indications of organ maladies or impairments; (5) professionally acquired informed permission was given by the patients or their families, signifying their voluntary participation in the study and their agreement to sign the informed consent form.

### 2.2. Chemotherapy and Immunochemotherapy Regimens

Patients were treated with a 21-day cycle of chemotherapy and anti-PD-1 monoclonal antibodies (mAbs). The anti-PD-1 mAb substitutes, which were administered intravenously on the initial day of every treatment cycle, were 200 mg of sintilimab, 200 mg of camrelizumab, and 200 mg of nivolumab. The chemotherapy regimens in this study consisted of the following protocols: (1) oxaliplatin and capecitabine, on the 1st day of the cycle, an intravenous dose of 130 mg/m^2^ of oxaliplatin was given. Additionally, capecitabine was given orally with a dose of 1,000 mg/m^2^ for 14 days, commencing on day 1 and taken twice daily. (2) For oxaliplatin and tegafur, an intravenous dose of 130 mg/m^2^ of oxaliplatin was given on the 1st cycle day. Additionally, tegafur was taken orally for 14 days at a dosage of 40 mg/m^2^, starting on the 1st day and requiring two tablets each day. (3) For tegafur with albumin-bound paclitaxel, on the 1st and 8th days of the cycle, an intravenous dose of albumin-bound paclitaxel (120 mg/m^2^) was administered. Moreover, tegafur was taken twice a day for 14 days at a dose of 40 mg/m^2^, and oral administration of the medication started on the 1st day.

### 2.3. Efficacy Assessments

Patients with measurable lesions had their therapeutic response evaluated using the Response Evaluation Criteria in Solid Tumors version 1.1 (RECIST1.1) criteria [19]. A complete response (CR) was defined as the elimination of all target lesions, while a partial response (PR) was defined as the decrease of the total number of target lesions by more than 30%. Conversely, an increase of more than 20% in the total number of target lesions indicated progressive disease (PD), whereas a decline of less than 30% or a rise of less than 20% suggested stable disease (SD). The ratio of CR + PR + SD cases to all cases, with all criteria met for at least 4 weeks, was used to compute the disease control rate (DCR).

### 2.4. Blood Sampling Collection

In anticipation of the subsequent immunochemotherapy cycle, blood collection was timed to coincide with the conclusion of the current treatment regimen, just before the onset of the subsequent cycle. Using conventional venipuncture methods, samples of the entire blood were drawn and placed in serum extractor tubes. The serum was then separated from the tube contents by centrifugation at 4,000x gravity for 10 min. The separated serum was promptly stored at −80°C in preparation for further examination in the clinical lab.

### 2.5. Cytokine Assessment

An entirely automated chemiluminescence immunoassay analyzer (Biolihe, Suzhou, China) was used to test serum samples from each patient. In accordance with the manufacturer's instructions, we measured the blood levels of IL-2R, IL-10, IL-6, TNF-*α*, IL-1*β*, IL-8, and IL-17. Kit standards and Multiplex controls were used as assay controls in the five-parametric curve fitting data analysis. Technical oversight for these experiments was provided by Biolihe's trained personnel.

### 2.6. Statistical Analysis

GraphPad Prism 8.0 (GraphPad Software Inc.) was used for statistical analysis and the drawing of graphs. Nonparametric Mann–Whitney *U* test was employed for comparisons between two groups for measurement data. Correlation analysis was done using Pearson correlation analysis. In order to evaluate the prediction power of different diagnostic techniques, receiver operating characteristic (ROC) curves were created, employing a binary logistic regression model. Statistical analysis was performed using DeLong's test. A statistical significance threshold of *p* < 0.05 was employed to ascertain the significance.

## 3. Results

### 3.1. Patient Population

Between March 2022 and March 2023, an amount of 56 AGC patients who met the research inclusion criteria were seen at Jiangsu University's Affiliated Hospital. 36 patients diagnosed AGC (36/56) took immunochemotherapy regimen and 20 marched AGC patients who only received chemotherapy regimen without PD-1 blockade immunotherapy were used as the control group. Following the RECISTv1.1 standard, the disease control rate (DCR) among 36 AGC patients receiving PD-1 blockade immunochemotherapy was determined to be 69%. Among these, 25 patients were categorized as the benefited group, while 11 patients were classified as the not benefited group in terms of their response to immunochemotherapy. The DCR of AGC patients in only chemotherapy group (control) was 50%, respectively (Table 1).

### 3.2. Cytokine Levels in the Benefitted, Not Benefited, and Control Groups Are Compared

**Figure 1:**
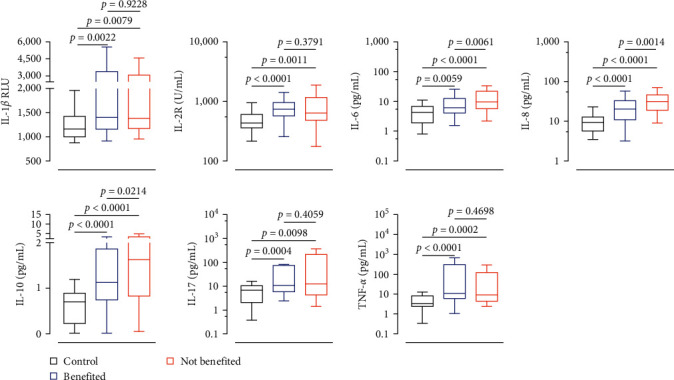
Comparison of serum cytokine concentrations in advanced gastric cancer (AGC) patients receiving chemotherapy (control) versus immunochemotherapy (benefited and not benefited groups). The histograms illustrate differences in cytokine profiles between the control group (*n* = 20) and patients in the benefited (*n* = 25) or not benefited group (*n* = 11). The median values are denoted by red bars. Statistical analysis for each pair of datasets was conducted using the Mann–Whitney *U* test, with significance set at *p* < 0.05.

To elucidate the cytokine modulation pattern following PD-1 blockade immunotherapy, serum cytokine levels were evaluated in control, benefited, and not benefited group. As demonstrated by Figure 1, there was a substantial difference in the presentation levels of IL-1*β*, IL-2R, IL-6, IL-8, IL-10, IL-17, and TNF-*α* between the control group (*n* = 20) and the benefited group (*n* = 25) (*p*=0.0022, *p* < 0.0001, *p*=0.0059, *p* < 0.0001, *p* < 0.0001, *p*=0.0004, and *p* < 0.0001, respectively) and markedly lower compared to those in the not benefited group (*n* = 11; *p*=0.0079, *p*=0.0011, *p* < 0.0001, *p* < 0.0001, *p* < 0.0001, *p*=0.0098, and *p*=0.0002, respectively), suggesting diminished cytokine levels in AGC patients treated solely with chemotherapy. Nonetheless, there were no discernible variations in the expression levels of IL-1*β*, IL-2R, IL-10, IL-17, and TNF-*α* between the groups that benefited and those who did not (*p*=0.9228, *p*=0.3791, *p*=0.0214, *p*=0.4059, and *p*=0.4698, respectively). Interestingly, compared to the not benefited group, the blood levels of IL-8 and IL-6 in the benefited group were much lower (*p*=0.0061, *p*=0.0014). These findings suggested that immunochemotherapy promotes cytokine release in AGC patients.

### 3.3. Dynamic Cytokine Alterations in Individuals with AGC during Immunochemotherapy

**Figure 2:**
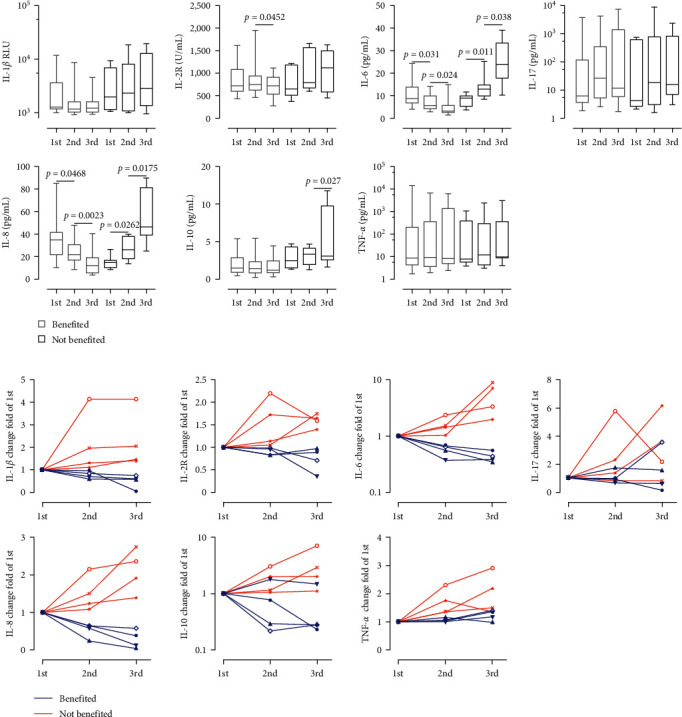
Dynamic changes in serum cytokine levels in AGC patients during immunochemotherapy. (a) Graphs depict IL-1*β*, IL-2R, IL-10, IL-6, IL-8, IL-17, and TNF-*α* levels at different time points in patients who benefited (*n* = 10) and did not benefit (*n* = 10) from immunochemotherapy. (b) Longitudinal graphs illustrate cytokine kinetics changes from the initial evaluation in benefited (*n* = 4) and not benefited (*n* = 4) patients. Statistical analysis was performed using the Mann–Whitney *U* test, with significance set at *p* < 0.05.

In our retrospective validation cohort, we conducted an analysis of cytokine levels across three cycles following the initiation of immunochemotherapy, allowing for a more comprehensive examination of on-treatment cytokine dynamics. Among patients who did not benefit from immunochemotherapy, we observed a progressive increase in the levels of IL-6, IL-1*β*, IL-8, and IL-2R, from the initial assessment, while the levels of TNF-*α*, IL-17, and IL-10 displayed inconsistent changes. On the contrary, those who derived benefits from immunochemotherapy exhibited a progressive decline in IL-6 and IL-8 levels, while the levels of TNF-*α*, IL-2R, IL-1*β*, IL-17, and IL-10 showed varying fluctuations, either increasing or decreasing from the baseline assessment (Figure 2(a)). To provide a more intuitive depiction of cytokine dynamics during treatment, we employed line graphs to illustrate the trajectory in typical cases. The visual representations unveiled a downward trajectory in IL-6 and IL-8 levels among responsive patients throughout the therapy duration, contrasting with the progressive elevation observed in nonresponsive individuals. But the trends for IL-2R, TNF-*α*, IL-1*β*, IL-10, and IL-17 were inconsistent and lacked statistical significance (Figure 2(a)). Collectively, according to our research, individuals with advanced gastric cancer who benefit from immunochemotherapy may possess decreased IL-6 and/or IL-8 blood levels.

### 3.4. Correlation Investigation of the Tumor Markers CEA, CA50, CA199, and CA724 with Blood Levels of IL-6 or IL-8

**Figure 3:**
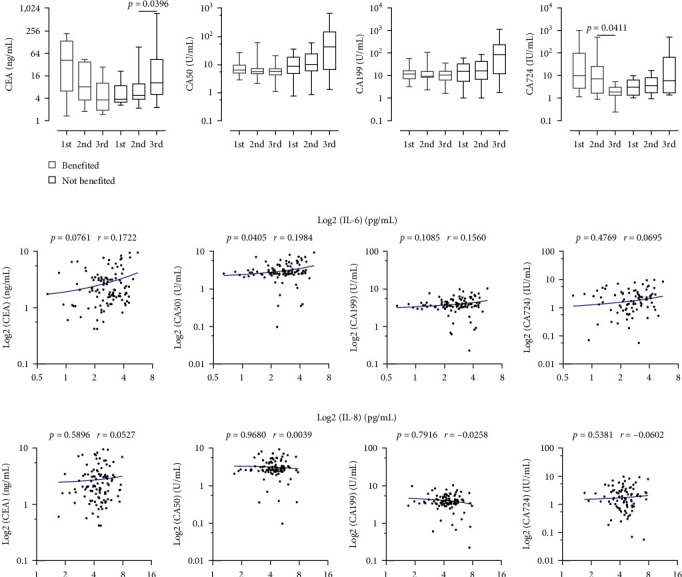
Correlation analysis of serum IL-6 and IL-8 with tumor markers in AGC patients undergoing immunochemotherapy. (a) Results of CEA, CA50, CA199, and CA724 levels plotted at different time points in the benefited (*n* = 25) and not benefited groups (*n* = 11). (b) Scatter plot showing the relationship between IL-6 level and CEA, CA50, CA199, and CA724 on all outcomes of three treatment courses for benefited (*n* = 25) and not benefited groups (*n* = 11). (c) The relationship between IL-8 and CEA, CA50, CA199, and CA724 on all outcomes of three treatment courses for benefited (*n* = 25) and not benefited groups (*n* = 11). Statistical analysis included the Mann–Whitney *U* test for each pair of datasets and Pearson correlation analysis to assess correlation; *p* < 0.05 indicates statistical significance.

Tumor markers serve as valuable biochemical indicators for predicting therapy efficacy. In our study, we analyzed GC-related tumor markers, including CEA, CA50, CA199, and CA724, in all patients throughout their therapy regimen. Our findings revealed a gradual increase in these tumor markers among patients who did not benefited from treatment (*n* = 10), while a gradual decrease was observed in those who experienced benefits (*n* = 10) (Figure 3(a)). To investigate the potential predictive role of cytokines and tumor markers in immunotherapy efficacy, we conducted a correlation analysis between blood IL-6/IL-8 concentrations and tumor markers in AGC patients receiving immunochemotherapy. Our findings revealed no significant correlation between serum IL-6 levels and CEA (*r* = 0.1722, *p*=0.0405), additionally between IL-6 and CA50 (*r* = 0.1984), CA199 (*r* = 0.1560), and CA724 (*r* = 0.0695) (Figure 3(b), *p* > 0.05), suggesting that IL-6 and tumor markers are independent detection indices. Furthermore, no meaningful relationship was discovered among serum IL-8 levels and CA724, CEA, CA50, or CA199 (Figure 3(c), all *p* > 0.05). Our findings suggested that IL-6 or IL-8 can be utilized as independent predictors of efficacy and complement tumor markers CEA, CA50, CA199, and CA724 in predicting the effectiveness of immunotherapy in AGC patients.

### 3.5. Serum IL-6 and IL-8 Were Coupled with Tumor Markers to Evaluate Clinical Improvements following Immunochemotherapy in AGC Patients

**Figure 4:**
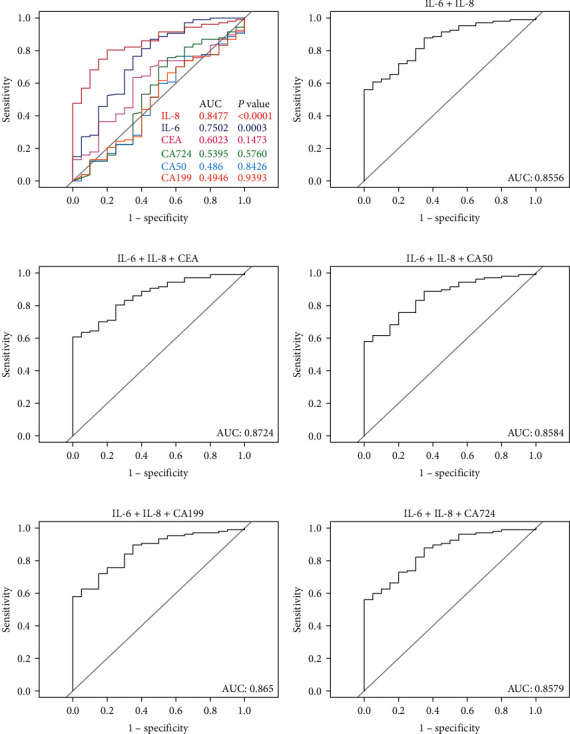
ROC curves for different marker combinations to evaluate clinical benefits in AGC patients undergoing immunochemotherapy. The AUC analysis was conducted for IL-6, IL-8, CEA, CA50, CA199, and CA724 alone (a) and in combination with IL-6 and IL-8 as described in (b–f). ROC curves were generated using the binary logistic regression model. Statistical analysis utilized the DeLong's test; *p* < 0.05 indicates statistical significance.

ROC curves are valuable tools for comparing the diagnostic performance of multiple screening tests for a given disease. Typically, the test with a higher area under the curve (AUC) is deemed superior. In our investigation into the synergistic relationship between IL-6/IL-8 and tumor markers for predicting the effectiveness of immunochemotherapy in AGC, ROC curves were used to evaluate the effectiveness of CA50, CA199, CEA, IL-6, IL-8, and CA724, as well as the combinations of these agents. The AUC values for IL-6, IL-8, CEA, CA724, CA50, and CA199 were determined as 0.7502 (*p*=0.0004), 0.8477 (*p* < 0.0001), 0.6023 (*p*=0.1473), 0.5395 (*p*=0.5760), 0.486 (*p*=0.8426), and 0.4946 (*p*=0.9393), respectively (Figure 4(a)). Results from the ROC analysis highlighted the superior predictive ability of IL-6 and IL-8 in forecasting the efficacy of immunochemotherapy. Subsequent analysis aimed to explore whether combining IL-6, IL-8, and tumor markers could enhance predictive power. The ROC curves for the combination of IL-6 and IL-8 yielded an AUC of 0.8556 (Figure 4(b)). Incorporating CEA, CA50, CA199, and CA724 into the IL-6 and IL-8 combination further elevated the AUC values (0.8724, 0.8584, 0.865, and 0.8579, respectively) (Figure 4(c)–4(f)). According to these findings, the assessment performance of IL-6, IL-8, and CEA in combination was better than that of each marker alone when it came to predicting the effectiveness of immunochemotherapy in AGC patients.

## 4. Discussion

Immune checkpoint blockade stands as a cornerstone in the therapeutic landscape for various cancers [20]. However, it is crucial to determine quickly whether the therapy is effective in the short term, as identifying responders and nonresponders can help avoid prolonged treatment and related toxicities and financial costs [11]. To tackle this challenge, alternative criteria, known as immune-related response criteria (irRC), have been devised [21]. However, irRC hinge on imaging evaluations, which harbor inherent limitations, particularly in the early assessment of treatment outcomes, defining response or progression proves challenging [22]. Presently, imaging assessments and tumor markers are customary in evaluating efficacy. However, they fall short as rapid evaluation markers for immunotherapy due to delayed responses and pseudoprogressions. Such unconventional response patterns impede the early differentiation of responders from nonresponders [23]. Recent experiences reveal that conventional response evaluation criteria underestimate the impact of checkpoint inhibitors due to delayed kinetics and atypical response patterns [24]. Conversely, while tumor markers exhibit sensitivity to therapeutic effects and are suitable for rapid immunotherapy evaluation, they also suffer from specificity issues and low positive rates. In essence, there are currently no methods to predict which patients may ultimately benefit from these immunotherapies at the early stage. Therefore, the discovery of novel predictive biomarkers is imperative to swiftly assess early efficacy in anti-PD-1/PD-L1 treatment. Given the release of abundant cytokines in responding individuals undergoing anti-PD-1/PD-L1 therapy due to the disinhibition of immunosuppressive responses, the dynamic changes in cytokines post-PD-1/PD-L1 blockade could rapidly reflect the initiation of an antitumor immune response in the body [25]. The alignment between these changes and clinical manifestations in tumor patients facilitates the objective discovery of new predictive markers.

Following this principle, our study employed imaging examinations, pathological assessments, and tumor markers as criteria to evaluate the effectiveness of PD-1 blocking therapy. Additionally, we conducted dynamic monitoring of blood cytokines in advanced GC patients to detect changes in characteristic cytokines. Our findings revealed that patients undergoing chemotherapy exhibited low baseline levels of all cytokines. However, those treated with immunochemotherapy displayed a global increase in cytokine levels following treatment initiation, indicating that immunochemotherapy can elicit a systemic immune response in both responsive and nonresponsive patients. A notable finding was that solely responsive patients displayed a declining pattern in IL-6 or IL-8 levels during the course of the therapy, a trend conspicuously absent in nonresponsive patients. This suggests that a characteristic that sets responsive patients apart from other patients is a decrease in IL-6 or IL-8 levels after treatment. The ability of IL-6 or IL-8 to discriminate between effective and ineffective treatment may be attributed to the nonspecific nature of PD-1 blockade. After receiving PD-1 antibody treatment, most tumor-related immune cells are activated, leading to an increase in various cytokines [26], as reflected in our results.

In responsive patients, tumor tissues gradually regress, and the highly activated state of the immune system diminishes, resulting in a gradual decline in IL-6 and IL-8 serum levels. Conversely, in nonresponsive patients, tumor progression persists, and the immune system remains highly activated, leading to a gradual increase in IL-6 and IL-8 blood levels. Consequently, IL-6 or IL-8 could be used as predictive indicators to quickly determine whether PD-1 blockage is effective in the short term. This conclusion partly aligns with recent reports [27–30], whereby prognosis of cancer patients is negatively correlated with elevated serum IL-8 expression levels, diminishes the clinical efficacy of immune checkpoint inhibitors, and can serve as an independent biomarker for patients receiving immune checkpoint inhibitors [31]. Additionally, our findings indicate that in responding individuals, IL-10 levels gradually decline, while TNF-*α* or IL-17 levels gradually rise, warranting further verification through subsequent large-scale studies.

Indeed, tumor markers in positive patients exhibit sensitivity to therapeutic effects and are suitable for the swift evaluation of immunotherapy. However, IL-6 or IL-8 did not show a significant correlation with tumor markers CEA, CA50, CA199, or CA724, suggesting partial independence and complementarity in predicting immunotherapy efficacy. Thus, we conducted an analysis to assess whether serum IL-6 and/or IL-8, in combination with tumor markers, could predict clinical benefits with high accuracy. Our findings demonstrated a remarkably high predictive ability to forecast clinical efficacy in patients with advanced gastric cancer receiving anti-PD-1 mAb treatment, especially when IL-6, IL-8, and CEA are combined (AUC: 0.872), underscoring the robustness of these biomarkers.

To summarize, our research highlights the possibility of using changes in blood levels of IL-6 and IL-8 as biomarkers to track and forecast the therapeutic advantages of PD-1 blockage in patients who have terminal GC. In clinical settings, assessing dynamic changes in serum IL-6/IL-8 levels combined with tumor markers through periodic measurements, typically 2–3 times after initiating therapy, could quickly and reliably forecast the effectiveness of treatment with anti-PD-1/PD-L1, especially when imaging evaluations yield inconclusive results. These biomarkers offer a noninvasive and sequential approach to monitoring the dynamic evolution of the antitumor immune response, providing clinicians with real-time feedback.

## Figures and Tables

**Table 1:** The clinicopathological parameters of patients.

Characteristics	Benefited (*N* = 25)	Not benefited (*N* = 11)	Control (*N* = 20)
Gender (*n*) (%)			
Male	15 (60%)	8 (73%)	12 (60%)
Female	10 (40%)	3 (27%)	8 (40%)
Age (year)			
⩽60	10 (40%)	5 (45%)	8 (40%)
>60	15 (60%)	6 (55%)	12 (60%)
Stage at diagnosis			
I–II	0	0	0
III–IV	25 (100%)	11 (100%)	20 (100%)
Histology			
Adenocarcinoma	24 (96%)	11 (100%)	20 (100%)
SCC	0	0	0
Unknown	1 (4%)	0	0
Differentiation			
Low	14 (56%)	8 (73%)	9 (45%)
Low medium	3 (12%)	1 (9%)	4 (20%)
Media	3 (12%)	0	3 (15%)
Medium high	1 (4%)	0	1 (5%)
High	0	0	0
Unknown	4 (16%)	2 (18%)	3 (15%)
PD-1 blockade			
Sintilimab	7 (28%)	4 (37%)	0
Camrelizumab	4 (16%)	2 (18%)	0
Nivolumab	14 (56%)	5 (45%)	0
Complete remission (CR)	1 (4%)	0	0
Partial remission (PR)	4 (16%)	0	1 (5%)
Stable disease (SD)	20 (80%)	0	9 (45%)
Disease progression (PD)	0	11 (100%)	10 (50%)

## Data Availability

Data are available from the corresponding author upon reasonable request.

## References

[1] Baxevanis C. N., Perez S. A., Papamichail M. (2009). Cancer immunotherapy. *Critical Reviews in Clinical Laboratory Sciences*.

[2] Wang T. W., Johmura Y., Suzuki N. (2022). Blocking PD-L1-PD-1 improves senescence surveillance and ageing phenotypes. *Nature*.

[3] Shitara K., Ajani J. A., Moehler M. (2022). Nivolumab plus chemotherapy or ipilimumab in gastro-oesophageal cancer. *Nature*.

[4] Banta K. L., Xu X., Chitre A. S. (2022). Mechanistic convergence of the TIGIT and PD-1 inhibitory pathways necessitates co-blockade to optimize anti-tumor CD8+ T cell responses. *Immunity*.

[5] Huang M.-Y., Jiang X.-M., Wang B.-L., Sun Y., Lu J.-J. (2021). Combination therapy with PD-1/PD-L1 blockade in non-small cell lung cancer: strategies and mechanisms. *Pharmacology & Therapeutics*.

[6] Wu Q., Qian W., Sun X., Jiang S. (2022). Small-molecule inhibitors, immune checkpoint inhibitors, and more: FDA-approved novel therapeutic drugs for solid tumors from 1991 to 2021. *Journal of Hematology & Oncology*.

[7] Morad G., Helmink B. A., Sharma P., Wargo J. A. (2021). Hallmarks of response, resistance, and toxicity to immune checkpoint blockade. *Cell*.

[8] Fowler A. M., Strigel R. M. (2022). Clinical advances in PET-MRI for breast cancer. *The Lancet Oncology*.

[9] Shimada H., Noie T., Ohashi M., Oba K., Takahashi Y. (2014). Clinical significance of serum tumor markers for gastric cancer: a systematic review of literature by the task force of the Japanese gastric cancer association. *Gastric Cancer*.

[10] Grunnet M., Sorensen J. B. (2012). Carcinoembryonic antigen (CEA) as tumor marker in lung cancer. *Lung Cancer*.

[11] Ji X., Dong A. (2022). FDG PET/CT in prostate metastasis from gastric cancer. *Clinical Nuclear Medicine*.

[12] Jiang Y., Jin C., Yu H. (2021). Development and validation of a deep learning CT signature to predict survival and chemotherapy benefit in gastric cancer: a multicenter, retrospective study. *Annals of Surgery*.

[13] Berry S., Giraldo N. A., Green B. F. (2021). Analysis of multispectral imaging with the AstroPath platform informs efficacy of PD-1 blockade. *Science*.

[14] Lheureux S., Matei D. E., Konstantinopoulos P. A. (2022). Translational randomized phase II trial of cabozantinib in combination with nivolumab in advanced, recurrent, or metastatic endometrial cancer. *Journal for Immunotherapy of Cancer*.

[15] Jiang C., Wang Y., Hu Q. (2020). Immune changes in peripheral blood and hematoma of patients with intracerebral hemorrhage. *The FASEB Journal*.

[16] Borish L. C., Steinke J. W. (2003). Cytokines and chemokines. *Journal of Allergy and Clinical Immunology*.

[17] Sellars M. C., Wu C. J., Fritsch E. F. (2022). Cancer vaccines: building a bridge over troubled waters. *Cell*.

[18] Oken M. M., Creech R. H., Tormey D. C. (1982). Toxicity and response criteria of the Eastern cooperative oncology group. *American Journal of Clinical Oncology*.

[19] Hodi F. S., Hwu W.-J., Kefford R. (2016). Evaluation of immune-related response criteria and RECIST v1.1 in patients with advanced melanoma treated with pembrolizumab. *Journal of Clinical Oncology*.

[20] Kubli S. P., Berger T., Araujo D. V., Siu L. L., Mak T. W. (2021). Beyond immune checkpoint blockade: emerging immunological strategies. *Nature Reviews Drug Discovery*.

[21] Eisenhauer E. A., Therasse P., Bogaerts J. (2009). New response evaluation criteria in solid tumours: revised RECIST guideline (version 1.1). *European Journal of Cancer*.

[22] Cottrell T. R., Thompson E. D., Forde P. M. (2018). Pathologic features of response to neoadjuvant anti-PD-1 in resected non-small-cell lung carcinoma: a proposal for quantitative immune-related pathologic response criteria (irPRC). *Annals of Oncology*.

[23] Saâda-Bouzid E., Defaucheux C., Karabajakian A. (2017). Hyperprogression during anti-PD-1/PD-L1 therapy in patients with recurrent and/or metastatic head and neck squamous cell carcinoma. *Annals of Oncology*.

[24] Hoos A., Eggermont A. M., Janetzki S. (2010). Improved endpoints for cancer immunotherapy trials. *JNCI Journal of the National Cancer Institute*.

[25] Taube J. M., Galon J., Sholl L. M. (2018). Implications of the tumor immune microenvironment for staging and therapeutics. *Modern Pathology*.

[26] Liu L. L., Skribek M., Harmenberg U., Gerling M. (2023). Systemic inflammatory syndromes as life-threatening side effects of immune checkpoint inhibitors: case report and systematic review of the literature. *Journal for Immunotherapy of Cancer*.

[27] Ryan B. M., Pine S. R., Chaturvedi A. K., Caporaso N., Harris C. C. (2014). A combined prognostic serum interleukin-8 and interleukin-6 classifier for stage 1 lung cancer in the prostate, lung, colorectal, and ovarian cancer screening trial. *Journal of Thoracic Oncology*.

[28] Schalper K. A., Carleton M., Zhou M. (2020). Elevated serum interleukin-8 is associated with enhanced intratumor neutrophils and reduced clinical benefit of immune-checkpoint inhibitors. *Nature Medicine*.

[29] An H. J., Chon H. J., Kim C. (2021). Peripheral blood-based biomarkers for immune checkpoint inhibitors. *International Journal of Molecular Sciences*.

[30] Laino A. S., Woods D., Vassallo M. (2020). Serum interleukin-6 and C-reactive protein areassociated with survival in melanoma patients receiving immune checkpoint inhibition. *Journal for Immunotherapy of Cancer*.

[31] Ohata Y., Harada T., Miyakoda H., Taniguchi F., Iwabe T., Terakawa N. (2008). Serum interleukin-8 levels are elevated in patients with ovarian endometrioma. *Fertility and Sterility*.

